# Ludwig van Beethoven—a psychiatric perspective

**DOI:** 10.1007/s10354-021-00864-4

**Published:** 2021-08-02

**Authors:** Andreas Erfurth

**Affiliations:** 1 1st Department of Psychiatry and Psychotherapeutic Medicine, Klinik Hietzing, Wolkersbergenstraße 1, 1130 Vienna, Austria; 2 Medizinische Universität Wien, Vienna, Austria

**Keywords:** Diagnosis, Classification, Alcohol use disorder, Bipolar spectrum, Temperament, Diagnose, Klassifikation, Alkoholkonsumstörung, Bipolares Spektrum, Temperament

## Abstract

Biographical accounts of famous artists usually try to relate the life story to the works (and vice versa). This gives the work a special “colour”, often the context for understanding for today’s recipients. This interrelation is complex and often judgmental, sometimes manipulative. Thus, medical (including psychiatric), characterological and psychodynamic assessments and interpretations must be made with great caution. Primary sources may be scanty and diagnostic concepts may have changed (Mozart died of *hitzigem Frieselfieber* [prickly heat fever]; in Hölderlin’s lifetime, schizophrenia or bipolar disorder did not yet “exist”). The attempt at a diagnostic classification often says more about the author and his time than about the artist (for example, the assessment of Robert Schumann’s or Friedrich Hölderlin’s mental illness). Against this background, elements of Ludwig van Beethoven’s biography are presented from a psychiatric perspective. In summary, Beethoven can be diagnosed with an alcohol use disorder. A pronounced hyperthymic temperament is likely to have had a clearly positive influence on the course of the disorder. In particular, no influence of the alcohol use disorder on the musical quality of the work can be proven. A clear episodic course of affective symptoms as in bipolar disorder is not demonstrable. The deafness caused a severe reduction in quality of life.

## Abstract (de)

Biographische Darstellungen berühmter Künstler versuchen in der Regel, die Lebensgeschichte mit den Werken (und umgekehrt) in Beziehung zu setzen. Das gibt dem Werk eine besondere „Farbe“, dem heutigen Rezipienten oft den Kontext zum Verständnis. Diese Wechselbeziehung ist komplex und häufig wertend, manchmal manipulierend. Somit sind medizinische (einschließlich psychiatrische), charakterologische und psychodynamische Einschätzungen und Deutungen mit großer Vorsicht vorzunehmen. Die Primärquellen können dürftig sein, die diagnostischen Konzepte können sich verändert haben (Mozart starb an „hitzigem Frieselfieber“; zu Hölderlins Lebzeiten „gab“ es noch keine „Schizophrenie“ oder „bipolare Störung“); der Versuch einer diagnostischen Einordnung sagt oft mehr über den Autor und seine Zeit aus als über den Künstler (etwa die Bewertung der psychischen Erkrankungen Robert Schumanns oder Friedrich Hölderlins). Vor diesem Hintergrund werden Elemente der Biographie Ludwig van Beethovens aus psychiatrischer Sicht dargestellt. Zusammenfassend lässt sich bei Beethoven eine Alkoholkonsumstörung diagnostizieren. Ein ausgeprägtes hyperthymes Temperament dürfte einen deutlich positiven Einfluss auf den Krankheitsverlauf gehabt haben. Insbesondere lässt sich kein Einfluss der Alkoholkonsumstörung auf die musikalische Qualität des Werks nachweisen. Ein klarer episodischer Verlauf affektiver Symptome wie bei der bipolaren Störung ist nicht nachweisbar. Die Taubheit verursachte eine starke Einschränkung der Lebensqualität.

## Introduction

This manuscript is the transcript of a presentation given at the meeting “Medical Humanities: Politzer 100—Beethoven 250—Raffael 500” organised for the Natural History Museum Vienna (*Naturhistorisches Museum*), the Vienna Health Group (*Wiener Gesundheitsverbund*) and the Work Group on Medical Humanities of the Austrian Academy of Sciences by Herwig Swoboda, Eduard Winter and Felicitas Seebacher in the *Narrenturm* in Vienna, October 16, 2020.

*Das heute die öffentliche Meinung dominierende Beethoven-Bild ist, 250 Jahre nach der Geburt des Komponisten, zu großen Teilen ein Fabrikat aus unermüdlich sich selbst reproduzierenden Stereotypen, aus deren Rückkopplungszirkel offenbar selbst auf der Basis aktueller Forschung kaum ein konsensfähiger Ausweg zu finden ist. (Hans-Joachim Hinrichsen, 2019 [* 1*]).*

The image of Beethoven that dominates public opinion today, 250 years after the composer’s birth, is to a large extent a fabrication of tirelessly self-reproducing stereotypes, from whose feedback loop it is apparently almost impossible to find a consensual way out, even on the basis of current research. (Translation by AE)

It is not the aim of this presentation to give a comprehensive review of Beethoven’s biography or to discuss all possible aspects of mental health (and eventually disorder) in his life, but rather to discuss some aspects of how psychiatry in general assesses psychopathological phenomena, including full-blown psychiatric disorder, and how, herewith, the perspective of a clinical psychiatrist could possibly contribute to the understanding of Ludwig van Beethoven and of his “image” beyond self-reproducing stereotypes.

## About perspectives

Biographical representations of famous artists usually seek to relate the life story to the works (and vice versa). This gives the work a special “colour”. Often it is this context that makes the works of art more accessible to today’s recipient. This setting in relation is complex and often judgmental, sometimes manipulative.

These manipulations can occur unintentionally, but in many cases they are deliberate. The cultural policy of the National Socialists, particularly during World War II, misused composers and their music by building a specific perspective. A good example of this cultural perspective “committed to the idea of race and nation” [2] and to the personality cult [3] is the 1942 Austrian movie “Whom the Gods Love” (German original: *Wen die Götter lieben*), in which Wolfgang Amadé Mozart is portrayed as a “God-gifted” and thus as a transfigured, outstanding personality. (Interestingly, in 1944, a “God-gifted list” [*Gottbegnadetenliste*] was created to identify for the present day such outstanding individuals—who were consecutively exempted from military service—including Richard Strauss, Carl Orff, Wilhelm Furtwängler and Herbert von Karajan). In 1939, the Vienna Philharmonic Orchestra started the tradition of a New Year’s concert, initially to strengthen the *Volksgemeinschaft* through cheerful “light” music, later to improve morale during war [4]. Again, a single composer (Johann Strauss II) was identified and portrayed as being outstanding. (Indeed, too many composers of light music were of Jewish origin, and therefore banned: Jacques Offenbach, Emmerich Kálmán, Leo Fall, Oscar Straus, Fritz Kreisler, Friedrich Hollaender. Interestingly, Johann Strauss II’s father, Johann Strauss I, according to racial laws should have been considered as “quarter Jew”, but this information was suppressed by the National Socialist cultural policy [3].)

In the context of personality cult (“Führer”-cult [5]) and transfigured, “God-gifted” composers, Beethoven was easily manipulated into the perspective of an Aryan genius, a German “titan”, a super-resilient hero able to transcend his deafness and create immortal art. Furthermore, Beethoven was presented as a “revolutionary” in a “new era” and thus in line with the presentation of the National Socialist German Workers’ Party as a revolutionary movement [6]. Post-war approaches to culture were not infrequently contaminated by the ideas of personality cult. The “titan” approach to Beethoven remained plausible, interpretations of Beethoven’s orchestral works by conductors such as Karajan with his “big”, “very lush and rich” sound [7] were popular, although alternatives were available, but clearly less successful (Hermann Scherchen, René Leibowitz). Only recently, the historically informed performance movement (e.g., Roger Norrington, Christopher Hogwood, John Eliot Gardiner) and the next generation of musicians has tried to find a new perspective to Beethoven’s works.

Clearly, the relation between listener expectations and adequate performance remains complicated [8]. But, particularly in Beethoven’s case, knowledge of historic perspectives is helpful. Today, probably, the risk of ideological misappropriation is smaller than the risk of trivialization serving economic interests. To give an example, just in time for the anniversary year 2020, a new book by the German author, Christine Eichel, was portrayed with the following words on the homepage of the author:

*Er ist der Rockstar seiner Zeit: schwierige Kindheit, rebellisches Künstlertum, provokatives Auftreten – dennoch wird er von allen bejubelt. Wohltemperiert ist so gar nichts an diesem Komponisten, der Freund und Feind düpiert, der die richtigen Leute kennt und die falschen Frauen liebt.*

*Das Buch „Der empfindsame Titan – Ludwig van Beethoven im Spiegel seiner wichtigsten Werke“ erzählt die fesselnde Geschichte eines Nonkonformisten. Ebenso informativ wie unterhaltsam führt es in den geistigen Kosmos Beethovens, berichtet von Liebeskomplikationen und bizarren Launen, von notorischen Geldnöten und eruptivem Humor. Jenseits gängiger Mythen wird Beethoven auf neue Weise erfahrbar … [* 9*].*

He is the rock star of his time: difficult childhood, rebellious artistry, provocative manners—yet he is acclaimed by everyone. Nothing at all about this composer, who deceives friend and foe, who knows the right people and loves the wrong women, is well-tempered.

The book “The Sentimental Titan—Ludwig van Beethoven in the mirror of his most important works” tells the intriguing story of a nonconformist. As informative as it is entertaining, it leads into the spiritual cosmos of Beethoven, reports on love complications and bizarre whims, notorious financial difficulties and eruptive humor. Beethoven can be experienced in a new way beyond established myths ... (Translation by AE)

## Assessments and diagnostic concepts in clinical psychiatry

How can the psychiatrist proceed in assessing a patient? The psychiatrist can observe symptoms [10] and thereafter unite them into a syndrome [11]. Then it can be checked whether the patient meets the criteria for a defined disorder [12, 13]. Unfortunately, with the exception of neurosyphilis, common psychiatric diagnoses (such as depression, bipolar disorder, schizophrenia) cannot be assigned causally, but are the expression of a clinical definition. These definitions can change over time. Panic disorders, posttraumatic stress disorders or borderline personality disorders are fairly new concepts, even the concept of schizophrenia is no older than 120 years, while people who meet today’s criteria for schizophrenia obviously existed before 1900. Therefore, it is difficult to retrospectively label people with modern diagnoses that did not exist during their lifetime (during Hölderlin’s lifetime there was no schizophrenia or bipolar disorder). The attempt at a diagnostic classification often says more about the author and his time than about the artist (for example, the assessment of Robert Schumann’s [14–18] or Friedrich Hölderlin’s [19, 20] mental illnesses or the assessment of the diagnoses of fictional characters such as Lucia di Lammermoor [21, 22]). In addition, diagnostic concepts of the past may be difficult to interpret today (Mozart, for example, died of *hitzigem Frieselfieber* [prickly heat fever] [23]). To summarise and in other words:

*Klinische Beobachtungen sind, wie alle andere Beobachtungen, Interpretationen im Licht der Theorien. (Karl R. Popper [* 24*])*

Clinical observations, like all other observations, are interpretations in the light of theories. (Translation by AE)

In addition, a retrospective assessment (or in general an assessment without personal examination) is difficult. Karl Ludwig Bonhoeffer (1868–1948)—in the context of a possible psychiatric disorder of Adolf Hitler—commented on this problem as follows:

*mußte ich sagen, daß für den Psychiater im allgemeinen der Grundsatz gilt, sich über den Geisteszustand eines lebenden Menschen nur dann verantwortlich zu äußern, wenn man ihn selbst untersucht oder mindestens gesprochen hat. Berichte von Dritten können natürlich wichtige, unter Umständen entscheidende diagnostische Hinweise enthalten; aber die Erfahrung lehrt, daß die psychiatrische Untersuchung gelegentlich ein überraschend anderes Bild ergibt, als es nach der Darstellung der Umgebung erscheint *[25].

I had to say that for the psychiatrist in general the principle applies that he should only express himself responsibly about the state of mind of a living person if he has examined the person himself or at least spoken. Third party reports can, obviously, contain important, possibly crucial diagnostic information; but experience shows that the psychiatric examination sometimes gives a surprisingly different picture than what appears after the description by the vicinity. (Translation by AE)

The attitude of the psychiatrist and psychoanalyst Otto Kernberg (*1928)—in the context of a possible psychiatric disorder of Donald Trump—is similarly cautious:

*Ich bin dagegen, Diagnosen bei noch lebenden politischen Persönlichkeiten zu stellen, die man nicht in der eigenen Praxis gesehen hat. … Was gegen solche öffentliche Ferndiagnosen spricht, ist aber vor allem, dass man nie sicher sagen kann, inwieweit gewisse öffentlich zur Schau gestellte Eigenschaften vorgespielt werden, um einen politischen Effekt zu erreichen. Deswegen kann man nie genau wissen, inwieweit so eine öffentliche Person im intimen Leben wirklich die Züge zeigt, die eine Diagnose rechtfertigen würden* [26].

I am against making diagnoses of living political personalities whom one has not seen in one’s own practice. ... What speaks against such public remote diagnoses, however, is above all that one can never say for sure to what extent certain publicly exhibited characteristics are being simulated in order to achieve a political effect. That is why you can never know exactly to what extent such a public person really shows the traits in his intimate life that would justify a diagnosis. (Translation by AE)

Kernberg then continues:

*Dagegen macht es mir nichts aus, bei einer verstorbenen Persönlichkeit eine Diagnose zu stellen, bei der wirklich viel Information über das Privatleben vorliegt, aus der man dann auch gute Schlüsse über ihr Leben, ihre Beziehungen etc. ziehen kann.*

On the other hand, I don’t mind making a diagnosis of a deceased personality when there really is a lot of information about private life, from which one can then draw good conclusions about his life, his relationships, etc. (Translation by AE)

## Temperament, creativity and the bipolar spectrum

**Fig. 1:**
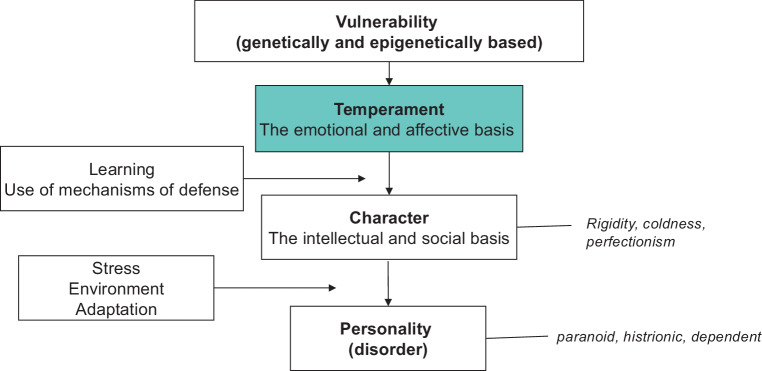
Definition of temperament as distinct from character and personality according to [27]

Temperament has been defined as the emotional and affective basis of our personality. Fig. 1 shows the position of the concept of temperament within human personality [27].

The perception that temperament [28, 29] and affective disorders are part of a continuum [27, 30] has strongly influenced modern psychiatry. While the typology of human temperaments can be traced back to Galen, the influential German psychiatrist Emil Kraepelin (1856–1926) described “basic states” (*Grundzustände*), that represent subaffective manifestations of “manic-depressive insanity” (*manisch-depressives Irresein*) [31, 32]: “*die leichteren und leichtesten Formen gehen ganz unmerklich in gewisse persönliche Eigentümlichkeiten über*” (“the lighter and lightest forms merge quite imperceptibly into certain personal peculiarities”, Translation by AE).

Today’s psychiatry acknowledges a continuum between subthreshold, “healthy” affective characteristics (hyperthymic temperament—with motivation and prominent performance capability as distinctive marks), cyclothymia [33, 34] and full-blown bipolar affective disorder with manic or hypomanic (and depressive) episodes [12, 13] and with or without characteristic comorbidities [35, 36].

**Fig. 2:**
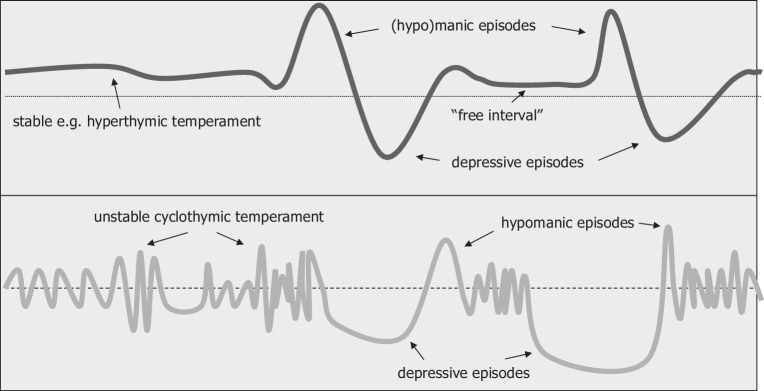
The pathoplastic model of temperament: intra-bipolar dichotomy according to [27]

Fig. 2 shows a pathoplastic model of temperament: depending on whether a stable (e.g., hyperthymic) or a cyclothymic temperament is present, the course of a bipolar disorder is modulated in two characteristic ways [27].

Particular attention has been given to the question of how temperament and bipolar diathesis interact with creativity [37–41]. This interaction is interesting in both directions, as music can be used to express psychopathology [21, 42] and as musicians can be affected by psychiatric disorders [42]. Recently, Greenwood has summarised the interaction of the bipolar spectrum with creativity as follows: “Bipolar disorder may thus be better conceptualised as a dimensional trait existing at the extreme of normal population variation in positive temperament, personality, and cognitive traits, aspects of which may reflect a shared vulnerability with creativity [43].”

## Beethoven, “revolutionary” and seducer of the masses in a “new era”?

With this knowledge of hypotheses and categories in psychiatry, we can now start to approach Ludwig van Beethoven from a psychiatrist’s perspective. The first question is: if Beethoven were a “revolutionary” in a “new era” (able to seduce masses until today), what do we know about the psychopathology of real revolutionaries, for instance in the Bavarian Council Republic [44]? Again, Karl Bonhoeffer is a good informant [25].

*Auch bei einer solchen, weite Volkskreise erfassenden psychischen Masseninfektion hat sich die Untersuchung auf die beiden Seiten zu erstrecken, die aktive führende Persönlichkeit und die psychische Zusammensetzung der geführten Masse. Wenn man sich an die nach unserer heutigen Erfahrung als relativ harmlos zu bezeichnende Revolutionswelle nach dem letzten Krieg im Jahre 1918/19 erinnert, so war es interessant, zu sehen, wie groß damals der Anteil psychopathischer Persönlichkeiten unter den führenden Männern der Räterepublik war. Es hat wohl kaum einen Psychiater gegeben, der nicht einen alten Bekannten aus seinen früheren Klinikinsassen plötzlich in irgendeiner führenden Stellung gesehen hat. … Eine sorgfältige, aus jener Zeit stammende klinische Untersuchung aus der Münchener Revolutionszeit ergab, daß es sich bei diesen psychopatischen Führerindividuen im wesentlichen um vier Typen gehandelt hat: ethisch Defekte, Hysterisch-Pseudologische, Fanatiker und Manisch-Depressive, zumeist von guter geistiger Begabung, gesteigerter Affektivität und Kritiklosigkeit gegenüber der eigenen Person und der übernommenen Aufgaben.*

Even in the case of such a mass mental infection, which affects large groups of people, the investigation must extend to both sides, the active leading personality and the mental composition of the mass led. If one recalls the wave of revolution after the last war in 1918/19, which according to our present experience could be described as relatively harmless, it was interesting to see how large the proportion of psychopathic personalities was among the leading men of the Council Republic at that time. There must have hardly been a psychiatrist who did not suddenly see an old acquaintance from among his former hospital inmates in some leading position.” ... “A careful clinical examination from the Munich revolutionary period from that time showed that these psychopathic leaders were essentially of four types: ethically defective, hysterical-pseudological, fanatic and manic-depressive, mostly of good intellectual talent, increased affectivity and lack of criticism towards their own person and towards the tasks taken on. (Translation by AE)

Obviously, this description does not apply to Beethoven, who, to the contrary, “was socialised in the Bonn court service, acted confidently in Viennese aristocratic circles and endeavoured to find various courtly positions until the end of his life” ([1], translation by AE).

## Beethoven and psychiatric diagnoses

While the importance of psychoanalytic contributions to the understanding of music in general [45–48] and the biography and works of Beethoven in particular [49–52] is acknowledged, the focus of the following is on a possible psychiatric diagnosis of Beethoven, notably the placement of Beethoven on the affective continuum between temperament and full-blown bipolar disorder.

As in other successful and productive people, numerous features of the hyperthymic temperament [31, 33, 35] can be found in Beethoven’s biography, namely increased energy and productivity, vividness, emotional intensity, resilience, tirelessness and strong will [56]. Hyperthymic temperament (risk taking, lack of anxiety) may facilitate the use of alcohol [53, 54], on the other hand, the social consequences of alcohol are less severe in hyperthymics [55]. Both aspects most likely apply to Beethoven.

To better understand what kind of person a famous artist was, it can be interesting to look at the encounter between two celebrities and at their mutual assessment (as described by Caeyers [56]). Such an encounter took place between July 19 and 26, 1812, in Teplitz (today Teplice, Czech Republic) between Ludwig van Beethoven (41 years old) and Johann Wolfgang von Goethe (62 years old).

Goethe wrote about Beethoven:

*Zusammengefasster, energischer, inniger habe ich noch keinen Künstler gesehen.*

I have never seen an artist more condensed, more energetic, more intimate.

*Ich begreife recht gut, wie er gegen die Welt wunderlich stehen muss.*

I understand quite well how he must stand wondrously against the world.

*Er ist leider eine ganz ungebändigte Persönlichkeit. Sehr zu entschuldigen ist er hingegen und sehr zu bedauern, da ihn sein Gehör verlässt, das vielleicht dem musikalischen Teil seines Wesens weniger als dem geselligen schadet. Er, der ohnehin lakonischer Natur ist, wird es nun doppelt durch diesen Mangel.*

He is unfortunately, quite an untamed personality. On the other hand, he is very much to be excused and very much to be pitied, since his hearing is leaving him, which is probably less detrimental to the musical part of his nature than to the sociable. He, who is of a laconic nature anyway, now becomes doubly so through this deficiency.

And Beethoven wrote about Goethe:

*Goethe behagt die Hofluft zu sehr, mehr als es einem Dichter ziemt. Es ist nicht viel mehr über die Lächerlichkeiten der Virtuosen hier zu reden, wenn Dichter, die als die ersten Lehrer der Nation angesehen sein sollten, über diesem Schimmer alles andere vergessen können.*

Goethe likes the court air too much, more than befits a poet. It is not much more to be said about the ridiculousness of virtuosos here, when poets, who should be regarded as the nation’s first teachers, can forget everything else above this gleam. (All translations by AE)

To sum up, Goethe describes Beethoven as “condensed, untamed, energetic and intimate”, confirming the main characteristics of the hyperthymic temperament. It is interesting that Beethoven was described by Goethe as laconic. While some hyperthymics can show joviality, talkativeness and tendency to repeat themselves, Beethoven clearly was different, at least on this particular occasion of meeting Goethe.

But what about full-blown affective (depressive or manic) episodes in Beethoven’s life as discussed by Davies [57] and Mai [58]? There are times of varying productivity [56], but the existence of clearly defined episodes, as with Robert Schumann [14–18] or Hugo Wolf [59], cannot be proven. The psychoanalysts Editha and Richard Sterba [60, 61] have dealt extensively with Beethoven’s conflict with his nephew Karl van Beethoven (1806–1858). Based on a psychoanalytical understanding of attachment and libido [62], they established the “polarity between the masculine and feminine principles” in Beethoven’s personality [63]. This “bipolarity” must not be confused with today’s definition for a bipolar disorder (formerly manic-depressive illness).

Kopitz [64] has discussed the possibility of borderline personality disorder. Beethoven’s biography, on the other hand, points less to cyclothymia, emotional instability and a disturbed identity than to a stable, hyperthymic personality [65] (for the concept of cyclothymia vs. hyperthymia see Fig. 2). Bower [66] believed “that between 1815 and 1820, Beethoven experienced a creative illness which was psychotic in type, ended in recovery and radically changed his musical creativity”. On the basis of the available sources, this cannot be reconstructed. Nor can any evidence be found of an anxiety disorder (such as in Charles Darwin [67]) or an obsessive-compulsive disorder (such as in Anton Bruckner [42, 68]).

The Heiligenstadt Testament [69, 70], a letter written by Beethoven to his brothers in 1802, has been linked to a depressive symptomatology. But while the letter clearly reflects the anguish of a 32-year-old man over his deafness, including a contemplation of suicide, it does not seem to be a document of depressive thought disturbance. It shows real and adequate despair and at the same time the attempt to overcome the consequences of this impediment.

## Does Ludwig van Beethoven meet today’s criteria for alcohol use disorder?

While full-blown affective or anxiety disorders were not found, the question remains whether Beethoven meets today’s criteria for alcohol use disorder. In this manuscript, the point has been made that it is difficult to retrospectively label people with modern “diagnoses” that did not exist during their lifetime. In the case of alcohol use disorder, the sources from Beethoven’s time can be used well, since the description of the behavioural and physical symptoms and consequences of alcohol use disorder is quite unambiguous and thus purposeful. Moreover, in 1852, only 25 years after Beethoven’s death, a concept of alcoholism was introduced (Magnus Huss. *Chronische Alkoholskrankheit oder Alcoholismus chronicus *[71]) that is quite compatible with modern ideas.

The American Psychiatric Association in its Diagnostic and Statistical Manual of Mental Disorders (fifth edition, DSM‑5 [12]) defines alcohol use disorder as follows:

A problematic pattern of alcohol use leading to clinically significant impairment or distress, as manifested by at least two of the following, occurring within a 12-month period:

1. Alcohol is often taken in larger amounts or over a longer period than was intended.
2. There is a persistent desire or unsuccessful efforts to cut down or control alcohol use.
3. A great deal of time is spent in activities necessary to obtain alcohol, use alcohol, or recover from its effects.
4. Craving, or a strong desire or urge to use alcohol.
5. Recurrent alcohol use resulting in a failure to fulfil major role obligations at work, school, or home.
6. Continued alcohol use despite having persistent or recurrent social or interpersonal problems caused or exacerbated by the effects of alcohol.
7. Important social, occupational, or recreational activities are given up or reduced because of alcohol use.
8. Recurrent alcohol use in situations in which it is physically hazardous.
9. Alcohol use is continued despite knowledge of having a persistent or recurrent physical or psychological problem that is likely to have been caused or exacerbated by alcohol.
10. Tolerance, as defined by either of the following:
  - A need for markedly increased amounts of alcohol to achieve intoxication or desired effect.
  - A markedly diminished effect with continued use of the same amount of alcohol.
11. Withdrawal, as manifested by either of the following:
  - The characteristic withdrawal syndromes for alcohol.
  - Alcohol is taken to relieve or avoid withdrawal symptoms.

Current severity is specified as follows:

Mild: Presence of 2–3 symptoms

Moderate: Presence of 4–5 symptoms.

Severe: Presence of 6 or more symptoms.

In matching biographical accounts [56, 69, 72] with DSM criteria [12], there is sufficient evidence to suggest that Beethoven meets criteria for (severe) alcohol use disorder, particularly for items 1, 2, 4, 9, 10 and 11.

Furthermore, there is good evidence [56, 69, 70] for the presence of a significant family history of alcohol use disorder. Ludwig’s grandfather, Ludovicus van Beethoven (1712–1773), served as Kapellmeister (director of music) in the Electoral court of Bonn and ran a wine business. His wife, Maria Josepha (about 1714–1775), was permanently institutionalised for alcohol use disorder at the end of her life. Their third child, Johann van Beethoven (1740–1792), became a singer at the Bonn court. In 1787, because of the severity of his alcohol use disorder, his oldest living son, Ludwig, at the age of 16 was entrusted with the care of his younger siblings and therefore received half of his father’s salary. Thus, Ludwig van Beethoven was clearly aware of the traumatic consequences of alcoholism at an early age.

As DSM‑5 [12] points out, “alcohol use disorder runs in families, with 40–60% of the variance of risk explained by genetic influences. The rate of this condition is three to four times higher in close relatives of individuals with alcohol use disorder, with values highest for individuals with a greater number of affected relatives, closer genetic relationships to the affected person, and higher severity of the alcohol-related problems in those relatives.” Unfortunately, all of these risk factors apply to Ludwig van Beethoven.

The course of alcohol use disorder is not uniform. The notion of alcohol use disorder as a permanently progressive disease eventually leading to social decline and death has proven to be wrong [73]. The influence of alcohol use on the biographies of many artists (such as writers E. T. A. Hoffmann, Paul Verlaine and F. Scott Fitzgerald, and composers Modest Mussorgsky, Jean Sibelius, Ernest John Moeran and Malcolm Arnold) is evident and shows a high degree of variety. It is difficult and ultimately futile to discuss whether Beethoven’s alcohol use disorder influenced his musical work. There is no evidence that alcohol use disorder could have affected the quality of Beethoven’s works. On the other hand, in the last months of his life—from late autumn 1826—the physical consequences (above all liver cirrhosis complicated by progressive ascites) [56, 69, 74–76] were certainly already so pronounced that they limited his ability to compose.

The physical consequences of alcohol use disorders are manifold. The DSM‑5 describes [12]: “Repeated intake of high doses of alcohol can affect nearly every organ system, especially the gastrointestinal tract, cardiovascular system, and the central and peripheral nervous systems. Gastrointestinal effects include gastritis, stomach or duodenal ulcers, and, in about 15% of individuals who use alcohol heavily, liver cirrhosis and/or pancreatitis.”

Ludwig van Beethoven died on 26 March 1827 at the age of 56. The original autopsy report [70, 77–79] of Beethoven’s remains indicates that he died of terminal liver cirrhosis [80, 81] with chronic pancreatitis, both of which appear to be related to his alcohol use disorder. The autopsy data also suggest that Beethoven had renal papillary necrosis and diabetes mellitus. Both can be associated with an alcohol use disorder. Alcohol use is one of the main causes of renal papillary necrosis. In addition, heavy drinking reduces the sensitivity to insulin, which can trigger type 2 diabetes [82], and chronic pancreatitis is a common cause of diabetes.

Hearing loss and ultimately deafness [70, 83, 84] can be the cause of mental disorders [85–88]; depressive withdrawal and paranoid thinking in people with hearing impairments are well documented, especially in the elderly. In Beethoven’s case, deafness was accompanied by impaired vision in the last years of his life [70]. Basically, however, there is no change of character due to the impairments. Despite the severe loss of quality of life, there is much to suggest that, given the circumstances, Beethoven remained sociable and without signs of paranoid processing until the end [56, 61].

In summary, from a psychiatric point of view, Beethoven can be diagnosed with alcohol use disorder. A pronounced hyperthymic temperament may have had a clear positive influence on the course of the disease. In particular, no effect of the alcohol use disorder on the musical quality of the work can be proven. A clear episodic course of affective symptoms as in bipolar disorder is not detectable. The deafness caused a severe reduction in quality of life.
